# Refusal of patients: care for people without health insurance in German emergency departments

**DOI:** 10.1186/s12910-024-01059-3

**Published:** 2024-05-16

**Authors:** Matthias Zimmer

**Affiliations:** Ketteler Hospital, Department of internal medicine I, Lichtenplattenweg 85, 63071 Offenbach, Hesse, Germany

## Abstract

In case of an emergency, health insurance in Germany provides easy access to medical care in emergency departments. Over 100,000 people do not have health insurance for various reasons. They are repeatedly refused treatment in emergency rooms as their right to care outside of regular insurance is often unknown or ignored.

German social law prescribes that every resident of the German state must have a health insurance. [Insurance Contract Act]. This insurance, which is provided either through statutory health insurance or private health insurance, offers general access to the healthcare system. This also includes the treatment of emergencies such as life-threatening illnesses, major injuries or severe pain. Emergency patients should be treated in emergency departments, which are used by 19 million patients every year [1]. In addition to critically ill patients, patients who are less seriously ill are also treated in the emergency departments [1]. Less seriously ill patients should be cared by the general practitioners, but many patients don’t want to accept this [2]. Due to acute emergency presentations, workload compression, and working hours, emergency department staff may experience stress regarding their mental and somatic health [2]. In this area of conflict, people without health insurance pose a particular challenge because familiar administrative procedures and medical matters are questioned.

According to the 2019 Microcensus, 61,000 people in Germany were reported to have no health insurance [3]. The number of unreported cases is probably much higher, estimated minimum 100,000 [4, 5], as the microcensus is a sample-based survey aimed at people with a permanent residence and a regular registration address [6, 7]. German language skills are also required to answer the questions. As a result, many people such as the homeless, migrants, people in precarious living situations etc. are not or not sufficiently covered. People of all ages are uninsured, tending to be younger than 50 years and have reduced health literacy [8–10]. Patients without health insurance cannot fulfill their obligation to have insurance because, among other things, they lack the financial means, their living situation is not described by the law, they are employed illegally or there are delays in the allocation of health insurance or equivalent state benefits. Anyone who does not have health insurance in Germany must pay for all medical services and use their own personal financial resources. But people without health insurance do not have the financial resources to pay for medical services, otherwise they would be able to afford health insurance [5, 8]. As a result, the need to maintain or restore one’s own health is not fulfilled.

In a few cities, people without health insurance can consult a charitable institution for medical care (Malteser Medizin für Menschen ohne Krankenversicherung, Ärzte der Welt, MediNetz). These alternative doctors’ offices don’t provide round-the-clock care and can’t take care of life-threatening emergencies. In principle, equipment at charitable institutions can rarely be compared with regular doctors’ offices. In emergency situations, uninsured or apparently uninsured patients find themselves in a dilemma. After all, alternative doctors’ offices are unable to care for them and, at the same time, charitable institutions report that these patients repeatedly experience refusals in emergency rooms.

This article results of intensive literature research based on the author’s professional experience as the director of an emergency department. The area of conflict between the economic and legal aspects of providing care to people without health insurance is highlighted. From this, possibilities are developed to find ways out of current dilemma.

## Economic aspects

In the German healthcare system, hospitals are managed similarly to commercial companies.

They are owned by the public sector (28.5%), non-profit companies (31.6%) and private companies (39.9%) such as limited companies, Societas Europaea or joint-stock companies [10]. In contrast to state or non-profit hospitals, private hospitals are highly profit-oriented companies. Returns can be up to 15% [11] Insured patients receive free health care in all hospitals, because health insurance cover medical costs. Patients with statutory health insurance have an insurance card with a smart card that identifies them as insured. Privately insured patients either have an insurance card or must provide their insurance details verbally to hospital administration.

In any case of medical care, uninsured patients generate the risk of a doubtful or irrecoverable debt in the respective annual balance sheet for the hospital. These missing earnings are not taken into account in the existing reimbursement system of diagnosis-related groups (DRG) [12]. For the period from 2011 to 2014, Mylinus showed that the annual uncovered costs of caring for uninsured migrants amounted up to 1.100.000 euros per hospital [13]. The type of ownership did not play a role here. In this context an individual physician, who is an employee of a company, cannot escape the economic pressure that has arisen in the healthcare system [14].

## Legal considerations

The Basic Law of the Federal Republic of Germany provides guidelines for social action under the Dictum of Human Dignity, Article 1 of the Basic Law [15]. The protection of dignity is also incumbent on physicians according to Sect. 7 of the Medical Professional Code [16]. To respect this and physical integrity [Article 2 of the Basic Law], it is an indispensable medical task to provide an indication for diagnosis or therapy [15]. The prerogative to make such decisions is based on a social contract [17], which places a high level of responsibility on the medical profession [16]. The advised curative intervention itself requires appropriate information derived from Section 630e of the German Civil Code [16, 18].

Legal framework requires direct contact between physicians and patients in Germany. Physicians are not the first contact for patients in emergency departments for organizational reasons, but rather administrative staff or nursing staff [19]. They could already refuse uninsured patients and not let them see a physician. The charitable organization Malteser Medizin für Menschen ohne Krankenversicherung (Malteser Medicine for People without Health Insurance) has gained corresponding experience with refused patients (Management meeting Malteser Medizin für Menschen ohne Krankenversicherung 01–02 June 2023, personal communication). A refusal without a physician’s involvement seems problematic from a legal point of view.

Physicians may refuse to treat a patient if there is no medical emergency (Sect. 7, Subsection 2 of the Professional Code of Conduct) [16]. In addition a physician can refuse treatment if there is no danger to life or severe pain and the patient is unable to pay for treatment [16].

However, in case of refusal by physicians further legal pitfalls must be considered. Hospital laws of the German federal states of Hesse [20] and Schleswig-Holstein [21] also stipulate that medically indicated treatment must be carried out regardless of the patient’s financial ability. Furthermore, requirements for failure to render assistance (Section 323c, German Criminal Code) should not be met [22]. This means not providing assistance in an emergency, even though it is necessary and can reasonably be expected [22]. The absence of an emergency is essential. However, bodily harm and manslaughter could also occur in conjunction with the legal concept of acting by omission if treatment is refused (Sects. 13, 212 and 223, German Criminal Code) [22]. As early as 1966, the German Federal Supreme Court ruled in a similar direction, holding physicians responsible for providing the best possible assistance within the scope of their abilities [23].

From a legal perspective, the refusal of a patient is associated with high hurdles and must be well justified. Only absence or apparent absence of health insurance is not a sufficient reason for a refusal.

## Legal situation of apparently uninsured patients

Patients who are apparently not covered by health insurance form a heterogeneous group consisting of homeless people, EU citizens, non-EU citizens, asylum seekers, war refugees and people with illegal residence status. Their legal rights differ significantly.

### Homeless German citizens

According to Social Code XII, social assistance offices (Sect. 3, Subsection 1, Twelfth Social Code) have to pay for healthcare (Sects. 47 to 52, Twelfth Social Code) for homeless people [24]. Depending on whether short-term help or longer-term help is required, the social assistance offices either cover the medical costs directly or indirectly via a health insurance fund as an intermediary (Sect. 98, Subsections 1 and 2, Twelfth Social Code) [24]. Physicians should have no concerns when treating homeless Germans, as medical costs are covered by social assistance offices.

### European union [EU]/ European economic community citizens [EEC]

For uninsured EU citizens and citizens of the EEC states regulations apply, in accordance with the Freedom of Movement Act/EU [25]. The European Court of Justice recently took the German government to task on this point [26] to provide assistance in case of illness, pregnancy, and maternity to non-German citizens who were actually residing in Germany (cf. Section 23, Twelfth Social Code) [24]. Again, responsible authorities are the social assistance offices. Social assistance is subordinate to other sources of benefits under Subsection 1 of Sect. 2, Twelfth Social Code [24], thus patients or hospitals must provide relevant evidence. The benefits are also not to be granted if the non-German citizens are not employees/self-employed persons, are in the first three months of their stay, are primarily looking for work, do not have a residence permit, have entered only for the purpose of receiving social assistance or are family members of the abovementioned individuals. According to Sect. 23, Subsection 3, Twelfth Social Code, people who do not have the right of residence in Germany can be granted a bridging allowance until they leave the country, at the longest for one month within two years [24]. This was recently confirmed by the 8th Senate of the Federal Social Court in summer 2023 [27]. Physicians in German emergency rooms should receive regular legal updates in addition to continuing medical education, as insurance law is constantly changing.

### Non-EU citizens

Non-EU citizens with a residence permit, according to the Residence Act [28], but without health insurance, come under the purview of Sect. 23, Twelfth Social Code in the same way as EU citizens [24]. According to Sect. 23 Twelfth Social Code, local social assistance offices must provide bridging assistance [24]. However, proof must be provided that no other sources of benefits exist in the sense of the subordination of social assistance pursuant to Sect. 2, Subsection 1, Twelfth Social Code [24]. It is difficult to imagine that hospitals have the capability to check sources of benefits of their patients. The implementation of state-financed clearing houses would be one way of relieving hospitals. Only very few clearing houses currently exist in Germany.

### Asylum seekers

People receiving benefits under the Asylum Seekers Benefits Act receive care for acute illness, pain conditions, and pregnancy, under Sect. 4 of the Residence Act [28, 29]. Even in the case of deportation healthcare is made possible within the framework of bridging benefits under Sect. 1, Subsection 4 of the Residence Act [28]. Depending on the federal state, the people concerned receive a health insurance card or apply for a treatment voucher from the concerned social assistance offices for each medical consultation. In the case of an acute emergency treatment certificate can also be applied for subsequently. Asylum seekers who have been in Germany for more than 18 months receive benefits under Sect. 2 of the Residence Act, analogous to those received by assistance recipients under Sects. 47–52 of the Twelfth Social Code [24, 28] Physicians should know that asylum seeker status could provide coverage for medical costs.

### War refugees

War refugees who arrive in the EU and, subsequently, in Germany as a result of a mass influx identified by the Council of the EU are granted a temporary right of residence [30]. Local social welfare authorities primarily assume the costs and subsequently pass them on to the federal government under Sect. 24, Residence Act [28]. The so-called “friction certificate,” which is issued by the local immigration authorities serves as proof. However, the temporary right of residence begins with the fulfillment of the criterion of seeking protection. The request for healthcare is already such a request for protection.

### People with illegal residence status

People who have crossed the German borders without an official residence permit (illegal residence status) can’t work legally and can’t take out health insurance. In the case of legal employment, registration with a health insurance is required. Again, health insurances are obliged to report their knowledge of a missing residence permit to immigration authorities. As a result, people with illegal residence would receive a fine or prison sentence and be deported from Germany [28]. If people with illegal residence and without health insurance come to an emergency department, it is possible that physicians refuse patients. Some physicians believe that they are liable to prosecution if they provide medical care to people with illegal residence status [31]. This assessment is clearly wrong according to §§ 95 and 96 of the Residence Act [28]. The German Federal Ministry of the Interior confirmed this in 2007 [32].

In principle, people living illegally can also receive emergency health benefits under Sect. 4 of the Asylum Seekers Benefits Act in conjunction with Sect. 48 of the Twelfth Social Code [24, 29]. However, because of the subordination of social assistance [Sect. 2, Subsection 1, Twelfth Social Code], it is difficult for the people concerned or the hospital to prove that they are in need [24]. Given the lack of documents in most cases of people living illegally, the required proof is difficult to maintain and is considered laborious by the hospital administrations [31]. In addition, major uncertainties exist regarding the transfer of data from hospitals to social assistance offices. This is because according to Sect. 87, Subsection 2 number 2a, Residence Act, public authorities have to inform immigration authorities if they become aware of people without a valid residence permit [28]. However, in the case of data originating from the doctor-patient relationship [see Sect. 203, Subsection 1 numbers 1, 2, 4, and 6, and Subsection 3, German Criminal Code], this should be prevented by the so-called “extended protection of secrets” [22, 33]. As this is only an administrative regulation and not a law, federal states interpret the regulations differently in some cases [31]. Therefore, any application for assistance could lead to a report to the immigration authorities. Emergency department staff should know what special protection applies to people with illegal residence status when it comes to health care. And they should know that the costs of emergency care are covered by the social assistance office.

## Hurdles in the administrative law

If a hospital wants to be paid for services rendered in retrospect (based on Sect. 4, Asylum Seekers’ Benefits Act in conjunction with Sect. 48, Twelfth Social Code) in the absence of a voucher for treatment, substantial entitlements are imposed on it [24, 29]. The hospital must immediately ask the social welfare agency by application whether the costs can be covered. If the authority cannot be reached, for example at night, the application must be submitted immediately on the next working day. Otherwise, the hospital’s claims expire.

## Medical ethos

The current multidimensional demands on physicians are enormous. Physicians should decide wisely [34]. The health of their patients should be the highest priority of their actions [35]. And they should work to safeguard or restore patient rights if these are violated [36].

In light of the economic pressure on the German healthcare system, physicians’ behavior should not be subordinated to optimizing the economic benefit of the hospital [37]. Simultaneously, physicians must bow to government-mandated business requirements. In sum, physicians also have expectations from themselves and their profession. Conflicting goals such as ethos versus framework conditions reduce physicians’ intrinsic motivation and, thus, their enjoyment of their work [38, 39]. Especially young physicians do not want to support primary monetary subordination of their actions [40]. If individual physicians try to combine all the abovementioned points as much as possible in their thinking and actions, they will carry a heavy burden due to high expectations. If hospital administrators rely on the rejection of patients when they are unable to pay for their treatment, physicians will face an ethical dilemma in conjunction with an enormous conflict of loyalty. This dilemma is exacerbated by the fact that younger physicians in the emergency departments are bound by instructions.

## Conclusions

The issue of people without health insurance comprises highly complex legal aspects. In some cases, there is only an apparent lack of health insurance and people really have various claims for cost coverage by the social assistance office. However, the actual insurance status can hardly be clarified ad hoc.

From a legal point of view, in Germany it is up to the physicians to decide whether the hospital may refuse a patient or not. A refusal must be made in accordance with the applicable law and should also include ethical considerations. In any case, a vital threat and consequential damage to the patient must be ruled out. Continuous legal training for physicians appears to make sense. Physicians receive rarely any legal teaching during their studies and specialist training, although their work is accompanied by numerous legal regulations. This creates uncertainty in decision-making. Directors of emergency departments and hospital administrations should define processes in accordance with medical ethics and legal situation in order to care for uninsured patients and clarify their true insurance status. This also includes the fact that the rejection of patients is a purely medical task that cannot be delegated to non-medical staff.

At the same time, legal hurdles should be reduced to make it easier to enforce existing legal entitlements to health care. Time requirements for hospitals to claim reimbursement should be made more flexible. In addition, defined reporting channels to the authorities would also be helpful in order to avoid delays in processing and loss of information. Implementation of state-financed clearing houses to clarify insurance claims could be helpful for apparently uninsured patients. Treatment funds could also be set up by the federal states to provide unbureaucratic health care in emergency situations. However, a reform of general hospital funding could also ensure that the financial pressure on physicians in their decision-making is reduced. All of these points can only be realized through social debate and political decision-making in parliaments.

## Data Availability

No datasets were generated or analysed during the current study.
